# Early Symptom Response in Patients With Uncomplicated Urinary Tract Infection Treated With Gepotidacin or Nitrofurantoin: Pooled Analysis From Two Pivotal Phase 3 Studies

**DOI:** 10.1093/cid/ciaf722

**Published:** 2026-01-06

**Authors:** Amanda J Sheets, Jeremy Dennison, Jason M Pogue, Keith S Kaye, Caroline Perry, Helen Millns, Jazmín Díaz-Regañón, Salim Janmohamed

**Affiliations:** GSK, Collegeville, Pennsylvania, USA; GSK, London, United Kingdom; College of Pharmacy, University of Michigan, Ann Arbor, Michigan, USA; Division of Allergy, Immunology and Infectious Diseases, and Department of Medicine, Rutgers Robert Wood Johnson Medical School, New Brunswick, New Jersey, USA; GSK, Collegeville, Pennsylvania, USA; GSK, Stevenage, United Kingdom; GSK, Madrid, Spain; GSK, London, United Kingdom

**Keywords:** urinary tract infection, gepotidacin, antibacterials, uncomplicated UTI

## Abstract

**Background:**

Uncomplicated urinary tract infections (uUTIs) can cause distressing symptoms seriously impacting daily activities. Early symptom relief during treatment, though fundamental to patients, is often overlooked in clinical trials. Gepotidacin, a new antibacterial agent, was noninferior to the first-line treatment nitrofurantoin in two randomized controlled trials (EAGLE-2 [NCT04020341] and EAGLE-3 [NCT04187144]). The current analysis utilized pooled data from these two trials to evaluate symptom responses during and after treatment in participants with uUTI.

**Methods:**

Participants were aged ≥12 years with at least two uUTI symptoms, urinary nitrite and/or pyuria, and no complicating factors. Participants were randomized 1:1 to oral gepotidacin (1500 mg) or nitrofurantoin (100 mg), both taken twice daily for 5 days. At baseline (Day 1), on-therapy (OT; Days 2–4), and test-of-cure (TOC; Days 10–13) visits, urinary symptoms were assessed (0–3; none–severe) from which a total score (0–12) was derived.

**Results:**

Overall, 3136 participants were randomized (1572 gepotidacin, 1564 nitrofurantoin). Mean (median) baseline symptom scores for gepotidacin and nitrofurantoin arms were 7.1 (7.0) and 7.2 (7.0), respectively; at OT, this decreased to 3.6 (3.0) for both arms. At the OT visit, >80% of participants had clinical improvement/resolution. Among those with baseline symptoms affecting everyday activities (moderate or severe), 53.2% (728/1369) assigned gepotidacin and 53.6% (738/1377) assigned nitrofurantoin reported at OT that symptoms no longer affected everyday activities (mild or absent); at TOC, these percentages were 93.8% (1232/1313) and 93.3% (1254/1344), respectively.

**Conclusions:**

Gepotidacin and nitrofurantoin provide meaningful and similar early relief from uUTI symptoms.

## Teaser

Symptoms of uncomplicated urinary tract infections can be severely debilitating; therefore, rapid symptom relief is important. EAGLE-2 and EAGLE-3 compared a new antibacterial, gepotidacin, with the first-line treatment nitrofurantoin. Symptom response analyses indicated that participants responded similarly early to both treatments.

Urinary tract infection (UTI) is a common condition, with a lifetime incidence in women of 50%–60% [1]. UTIs account for a substantial proportion of antibacterial prescriptions in the US primary care setting [2]. Generally, patients present with uncomplicated UTI (uUTI), defined as a lower UTI or acute cystitis in the absence of pregnancy, urinary tract abnormalities, and relevant comorbidities [3]. Symptoms of uUTI, (eg dysuria, frequency, and urgency) can be distressing, having a sizeable impact on patients’ quality of life and daily activities [4, 5]. Patient interview studies have confirmed symptom relief as the primary reason that patients seek UTI treatment [6, 7]; therefore, rapid symptom relief is crucially important.

Regulatory guidance for randomized controlled noninferiority trials in uUTI recommends that a composite (clinical and microbiological) primary efficacy endpoint be evaluated at a fixed timepoint post treatment in a microbiological comparator-susceptible analysis population [8, 9]. Thus, pivotal uUTI trials do not focus on symptom improvement shortly after initiation of treatment, or the impact of symptoms on daily activities and quality of life. This is despite such outcomes representing fundamental measures of therapeutic benefit to patients.

Observational studies investigating time to onset of uUTI symptom resolution have demonstrated that antibacterials reduce time to symptom resolution versus no antibacterial treatment [10, 11]. Fluoroquinolones have shown symptom improvement within 24 hours after treatment initiation [12, 13]. Fluoroquinolones are, however, no longer recommended for uUTI treatment due to the risk of rare serious adverse events and increasing resistance rates [14, 15]. In a small study of nitrofurantoin, a currently recommended first-line antibacterial for uUTI [3, 16], symptom improvement or resolution was achieved by Day 3 in 77% (27/35) of participants [17]. There is limited evidence from randomized controlled trials comparing early symptom responses for new antibacterial agents.

In two global, Phase 3, randomized, double-blind clinical trials (EAGLE-2 [NCT04020341] and EAGLE-3 [NCT04187144]), oral gepotidacin demonstrated noninferiority to nitrofurantoin based on combined clinical symptom resolution and microbiological eradication at the test-of-cure (TOC; Days 10–13) visit in the microbiological intent-to-treat nitrofurantoin-susceptible (micro-ITT NTF-S) populations [18]. Gepotidacin showed an acceptable safety profile with no life-threatening or fatal adverse events. Based on the results of EAGLE-2 and EAGLE-3, gepotidacin was approved in March 2025 by the US Food and Drug Administration (FDA) for the treatment of female adult and pediatric patients 12 years of age and older weighing at least 40 kg with uUTIs [19].

Using pooled EAGLE-2 and EAGLE-3 data, this analysis evaluated clinical responses and the daily impact of changes in symptom severity at the on-therapy (OT) visit (Days 2–4; ie, early clinical responses) and TOC visit among study participants in the intent-to-treat (ITT) population.

## METHODS

### Study Design

This analysis utilized pooled data from replicate noninferiority trials (EAGLE-2 and EAGLE-3) comparing oral gepotidacin to nitrofurantoin for treating uUTI [18]. The trials were conducted in accordance with the Declaration of Helsinki principles and the International Council for Harmonisation guidelines for good clinical practice. The protocols were approved by all necessary ethics committees and institutional review boards. All participants’ written consent was obtained.

Designed in accordance with the 2019 US FDA guidelines for uUTI and the current European Medicines Agency guidelines [8, 9], both trials randomized eligible female participants with uUTI 1:1 to receive twice-daily oral gepotidacin (1500 mg) or nitrofurantoin (100 mg), for 5 days. Eligible participants were aged ≥12 years, weighing ≥40 kg, with at least two symptoms of dysuria, frequency, urgency, or lower abdominal pain, as well as evidence of urinary nitrite and/or pyuria. Key exclusion criteria included genitourinary abnormalities; non-infectious urinary symptoms (such as overactive bladder, chronic incontinence, or chronic interstitial cystitis) that could confound symptom measurements and/or preclude uUTI resolution; upper UTI; or otherwise complicated UTI. Randomization was stratified by age group (<18 years, 18–50 years, and >50 years) and history of recurrent uUTI.

Participants attended a baseline visit on Day 1 (day of first dose), an OT visit on Days 2–4, a TOC visit on Days 10–13, and a follow-up visit on Days 25–31 (per protocol). During study participation through TOC, participants were prohibited from taking non-study medications or supplements that could interfere with clinical assessments of treatment response, including but not limited to nonsteroidal anti-inflammatory drugs. Further eligibility details and methodology have been published previously [18].

The ITT population comprised all randomized participants. Pre-defined clinically evaluable (CE) populations were defined prior to conducting the EAGLE-2 and EAGLE-3 studies, and comprised participants in the ITT population who followed study procedures per the protocol, including received planned treatment as randomized with ≥80% compliance; had evaluable symptom scores at baseline, OT (CE-OT) and TOC (CE-TOC); had not received other systemic antibacterials before the respective post-baseline visit unless it was for the current infection; and had no other major protocol deviations preventing evaluation of efficacy.

### Clinical Symptom Scores and Outcomes

**Figure 1.**
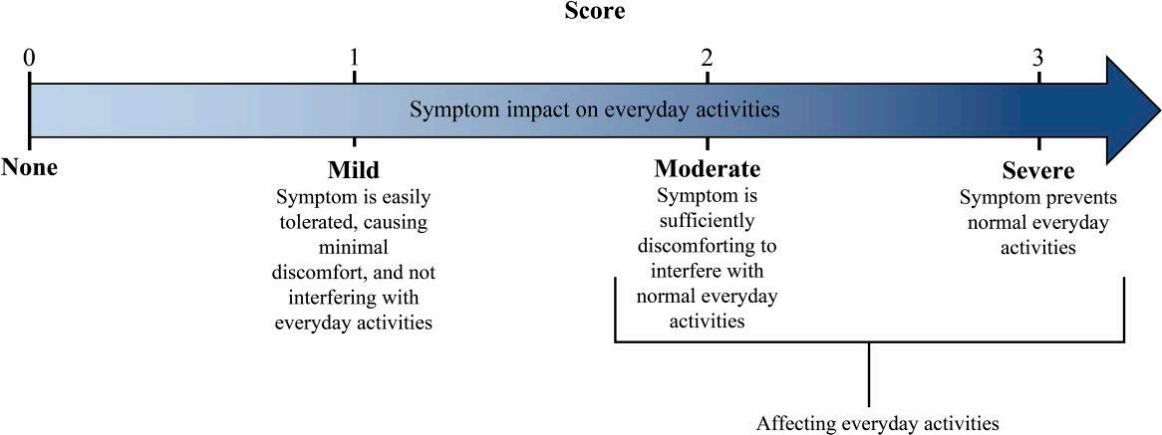
Symptom scoring in EAGLE-2 and EAGLE-3.

Individual symptom severity for dysuria, frequency, urgency, and lower abdominal pain was assessed at each study visit by participant interview and assigned a score of 0–3 (none, mild, moderate or severe) according to impact on daily activities (Figure 1). Total symptom scores (0–12) were programmatically derived based on the individual symptom scores, with a score of ≥2 required for study entry.

Clinical outcomes at OT and TOC were categorized as clinical resolution (total symptom score of 0 and without other antibacterials received prior to assessment), clinical improvement (reduction in total symptom score from baseline and without other antibacterials received prior to assessment), clinical worsening (increase/no change in total symptom score, and/or additional non-study antibacterials received for uUTI through the day of assessment), or unable to determine (due to missing data or receipt of non-study antibacterials, not for uUTI, that may have confounded efficacy assessments).

Participants with an outcome of “clinical resolution” or “clinical improvement” at the OT visit were considered early clinical responders for treatment comparisons. All other participants were considered non-responders at the OT visit. At the TOC visit, participants with an outcome of “clinical resolution” were considered a clinical success for treatment comparisons. All other participants were considered clinical failures at TOC, per regulatory guidance [8, 9].

A numeric symptom score threshold that represents clinically meaningful symptom relief has not been validated for uUTI. However, to evaluate symptom improvement that is potentially meaningful to patients, overall functional impact of improvement of urinary symptoms was assessed post hoc. Functional improvement of symptoms was defined as a participant's symptom scores all indicating no impact on everyday activities (ie, a mild [1] or absent [0] individual symptom) at OT and/or TOC having previously had any individual baseline symptom score indicating interference with and/or prevention of everyday activities (ie, a moderate [2] and/or severe [3] individual symptom) (Figure 1).

### Statistical Analyses

While the primary analyses in EAGLE-2 and EAGLE-3 used the interim analysis populations (as the studies were stopped early for efficacy), all analyses reported here were conducted in the complete, pooled ITT population; clinical outcomes were additionally evaluated in the pooled CE population. Outcomes were assessed using analysis visit windows as follows: OT (Days 2–5); TOC (Days 9–16). Data collected outside of these windows were considered missing, including for analyses of response by study day in which the visits were completed. The protocol-defined visit windows were widened for analysis to utilize out-of-window data considered consistent with the timepoint. Adjusted treatment differences (95% confidence interval [CI]) in response rates were calculated using the Miettinen–Nurminen (score) method, adjusting for study. Pooled analyses were not subject to hypothesis testing; results are descriptive.

## RESULTS

### Demographics and Other Baseline Characteristics

In total, 3136 participants with uUTI were randomized (pooled ITT population) in the EAGLE-2 and EAGLE-3 trials, with 1572 assigned to receive gepotidacin and 1564 assigned to receive nitrofurantoin. Demographics and baseline characteristics, including clinical symptom scores, were well balanced between the treatment groups (Table 1). Across treatment groups, the mean (standard deviation [SD]) uUTI symptom score was 7.2 (2.47). In total, 97% of participants reported urinary frequency, 93% had dysuria, 93% experienced urgency, and 82% had lower abdominal or suprapubic pain. Overall, 92% of participants reported one or more moderate to severe symptoms that interfered with or prevented daily activities (49% reported their worst symptom[s] was moderate and 43% had a severe symptom[s]; Supplementary Table 1). Overall, 8% of participants reported only mild symptoms that did not interfere with daily activities.

**Table 1.** Demographics and Baseline Characteristics (Pooled ITT Population)

	Gepotidacin 1500 mg BID n = 1572	Nitrofurantoin 100 mg BID n = 1564	Total N = 3136
*Age, years*			
Mean (SD)	48.9 (17.84)	49.4 (17.96)	49.1 (17.90)
<18	14 (<1%)	12 (<1%)	26 (<1%)
≥18–50	799 (51%)	799 (51%)	1598 (51%)
>50	759 (48%)	753 (48%)	1512 (48%)
51–64	404 (26%)	375 (24%)	779 (25%)
65–74	227 (14%)	229 (15%)	456 (15%)
≥75	128 (8%)	149 (10%)	277 (9%)
*Race*			
American Indian or Alaskan Native	64 (4%)	76 (5%)	140 (4%)
Asian	74 (5%)	86 (5%)	160 (5%)
Black or African American	114 (7%)	102 (7%)	216 (7%)
White	1301 (83%)	1289 (82%)	2590 (83%)
Other^a^	18 (1%)	11 (<1%)	29 (<1%)
Missing	1	0	1
*Ethnicity*			
Hispanic or LatinX	524 (33%)	502 (32%)	1026 (33%)
Not Hispanic or LatinX	1048 (67%)	1062 (68%)	2110 (67%)
*Geographic region*			
North America	957 (61%)	961 (61%)	1918 (61%)
United States	863 (55%)	867 (55%)	1730 (55%)
Europe	548 (35%)	523 (33%)	1071 (34%)
Asia-Pacific	67 (4%)	80 (5%)	147 (5%)
*BMI, kg/m^2^*			
Mean (SD)	27.4 (5.07)	27.1 (5.14)	27.3 (5.10)
<25	531 (34%)	570 (36%)	1101 (35%)
25–<30	576 (37%)	548 (35%)	1124 (36%)
≥30	465 (30%)	445 (28%)	910 (29%)
Missing	0	1	1
*Renal function, CrCl* ^b^			
Normal (≥90 mL/min)	1188 (78%)	1151 (76%)	2339 (77%)
Mild impairment (≥60 to <90 mL/min)	265 (17%)	267 (18%)	532 (17%)
Moderate (≥30 to <60 mL/min) or severe (<30 mL/min) impairment	76 (5%)	99 (7%)	175 (6%)
Missing	43	47	90
*History of diabetes*			
Yes	186 (12%)	197 (13%)	383 (12%)
No	1386 (88%)	1366 (87%)	2752 (88%)
Missing	0	1	1
*History of recurrent uUTI* ^c^			
Yes	646 (41%)	647 (41%)	1293 (41%)
No	926 (59%)	917 (59%)	1843 (59%)
*≥1 baseline uropathogen*			
≥10^3^ CFU/mL	1045 (66%)	1011 (65%)	2056 (66%)
≥10^5^ CFU/mL	732 (47%)	689 (44%)	1421 (45%)
*Clinical symptom scores*			
Mean (SD)	7.1 (2.50)	7.2 (2.44)	7.2 (2.47)
2–5	402 (26%)	389 (25%)	791 (25%)
6–8	706 (45%)	690 (44%)	1396 (45%)
9–12	462 (29%)	482 (31%)	944 (30%)
Missing	2	2	4
*Individual symptoms present (scores 1–3)* ^d^			
Dysuria	1474 (94%)	1443 (92%)	2917 (93%)
Frequency	1518 (97%)	1506 (96%)	3024 (97%)
Urgency	1463 (93%)	1453 (93%)	2916 (93%)
Lower abdominal or suprapubic pain	1269 (81%)	1295 (83%)	2564 (82%)

Data are n (%) unless otherwise stated; percentages were calculated with non-missing data as the denominator.

Abbreviations: BID, twice daily; BMI, body mass index; CFU, colony-forming units; CrCl, creatinine clearance; ITT, intent-to-treat; SD, standard deviation; uUTI, uncomplicated urinary tract infection.

^a^“Other” includes Native Hawaiian or other Pacific Islander, and Multiple.

^b^Based on the Cockcroft Gault formula.

^c^Recurrent infection was defined as a confirmed infection with at least one episode within the past three months, at least two episodes within the past six months, or at least three episodes within the past 12 m before study entry.

^d^One participant in the gepotidacin group and two participants in the nitrofurantoin group had missing baseline assessments for each symptom; one additional participant in the gepotidacin group had a missing baseline assessment for dysuria only. Total symptom score was considered missing if any individual symptom assessment was missing. Data are from post-hoc analyses.

### Clinical Outcome and Response by Visit

In the pooled ITT population, clinical outcomes at OT and TOC were similar between the gepotidacin and nitrofurantoin groups (Table 2). Most participants in both groups (>80%) had an early clinical response at OT. For gepotidacin and nitrofurantoin, clinical resolution was observed in 160 (10.2%) and 148 (9.5%) participants, respectively; clinical improvement (not including resolution) was observed in 1119 (71.2%) and 1136 (72.6%) participants at OT, respectively.

**Table 2.** Clinical Outcome and Response by Visit (Pooled ITT Population)

Visit Clinical Response Clinical Outcome	EAGLE-2 and EAGLE-3 Pooled Results		
	Gepotidacin 1500 mg BID n = 1572	Nitrofurantoin 100 mg BID n = 1564	Difference (95% CI)
*On-therapy*			
Early clinical response	1279 (81.4%)	1284 (82.1%)	–1.0 (–3.7, 1.6)
Clinical resolution	160 (10.2%)	148 (9.5%)	…
Clinical improvement (without resolution)	1119 (71.2%)	1136 (72.6%)	…
Non-response	293 (18.6%)	280 (17.9%)	…
Clinical worsening (or no change)	220 (14.0%)	212 (13.6%)	…
Unable to determine	73 (4.6%)	68 (4.3%)	…
*Test-of-cure* ^a^			
Clinical success (resolution)	1046 (66.5%)	1001 (64.0%)	2.5 (–.8, 5.9)
Clinical failure	526 (33.5%)	563 (36.0%)	…
Clinical improvement (without resolution)	347 (22.1%)	405 (25.9%)	…
Clinical worsening (or no change)	62 (3.9%)	76 (4.9%)	…
Unable to determine	117 (7.4%)	82 (5.2%)	…

Data are n (%) unless otherwise stated. Treatment difference (gepotidacin—nitrofurantoin) and 95% CI were calculated using the Miettinen–Nurminen method adjusted for study. Clinical outcomes for participants who took other systematic antibacterials for uUTI prior to, or on the same date as, their clinical assessment were set to “clinical worsening” (OT: 19 [1.2%] gepotidacin group, 17 [1.1%] nitrofurantoin group; TOC: 44 [2.8%] gepotidacin group, 62 [4.0%] nitrofurantoin group).

Abbreviations: BID, twice daily; CI, confidence interval; ITT, intent-to-treat; OT, on-therapy; TOC, test-of-cure; uUTI, uncomplicated urinary tract infection.

^a^Clinical outcomes at TOC for the individual study ITT populations have been reported previously [18].

The timing of post-baseline OT visits completed was similar between treatment groups (Supplementary Figure 1). The largest proportion of participants completed their OT visit on Day 2 (30%) or Day 3 (37%); 23% completed on Day 4, 5% on Day 5, and 5% did not complete the OT visit or the visit was out-of-window. Among those participants who completed the OT visit on Day 2, 73.7% (351/476) of the gepotidacin group participants and 71.8% (333/464) of nitrofurantoin group participants had an early clinical response (Supplementary Table 2). Among participants who completed the OT visit on Day 3, early clinical response was observed in 89.0% (512/575) of the gepotidacin group and 90.6% (528/583) of the nitrofurantoin group (Supplementary Table 2).

At TOC, clinical resolution was observed in 1046 (66.5%) participants assigned gepotidacin and 1001 (64.0%) participants assigned nitrofurantoin; clinical improvement (not including resolution) was observed in 347 (22.1%) and 405 (25.9%) participants, respectively (Table 2). Clinical outcomes were similar in the CE-OT and CE-TOC populations (Supplementary Table 3).

A post-hoc analysis showed that overall, 284 (9.1%) participants in the ITT population reported a medical history of urinary symptoms not related to a UTI in the past 12 months. A sensitivity analysis removing these participants showed similar clinical response results at OT and TOC in both treatment groups (Supplementary Table 4).

### Clinical Symptom Score Trends by Visit

**Figure 2.**
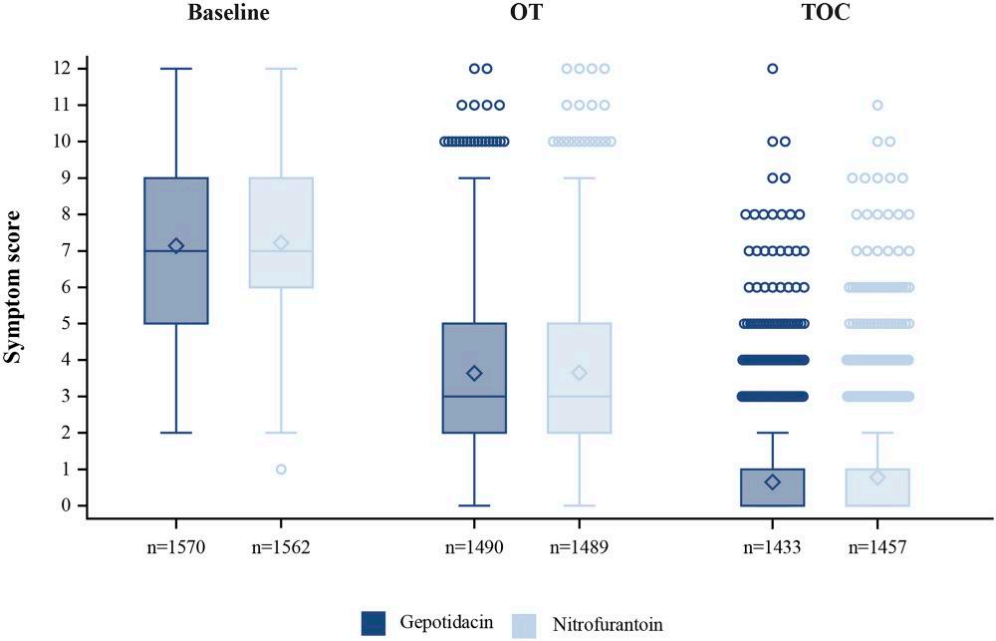
Total clinical symptom score by visit (pooled intent-to-treat (ITT) population).

**Figure 3.**
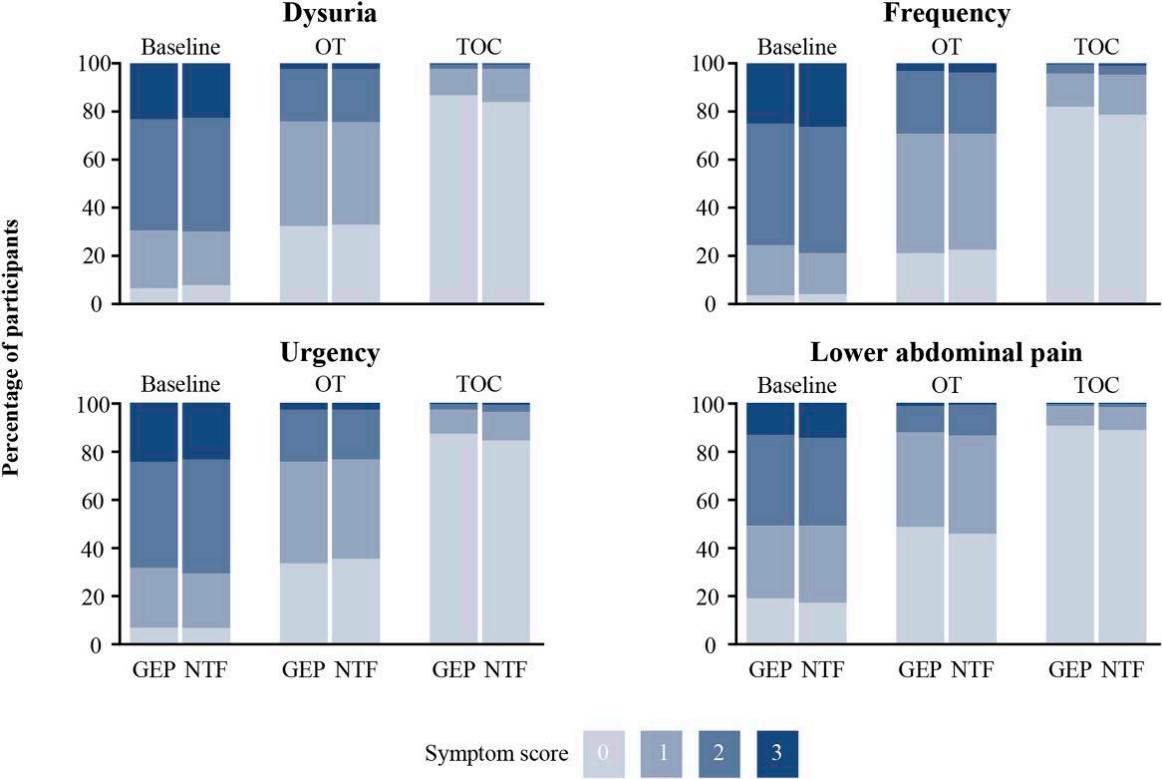
Individual clinical symptom scores by visit (pooled intent-to-treat (ITT) population).

Mean (median) total symptom score in the gepotidacin group decreased from 7.1 (7.0) at baseline to 3.6 (3.0) at OT and 0.6 (0.0) at TOC in the ITT population (Figure 2). Similar results were observed for nitrofurantoin: 7.2 (7.0) at baseline; 3.6 (3.0) at OT; 0.8 (0.0) at TOC. No notable differences in individual symptom severity scores were observed between treatment groups across visits (Figure 3). Rates of symptom improvement at OT and symptom resolution at TOC by individual symptom present at baseline were evaluated in a post-hoc analysis (Supplementary Table 5). Symptom relief was broadly similar across the individual symptoms in both treatment groups.

### Functional Improvement of Uncomplicated Urinary Tract Infections Symptoms Interfering With or Preventing Daily Activities by Visit

**Figure 4.**
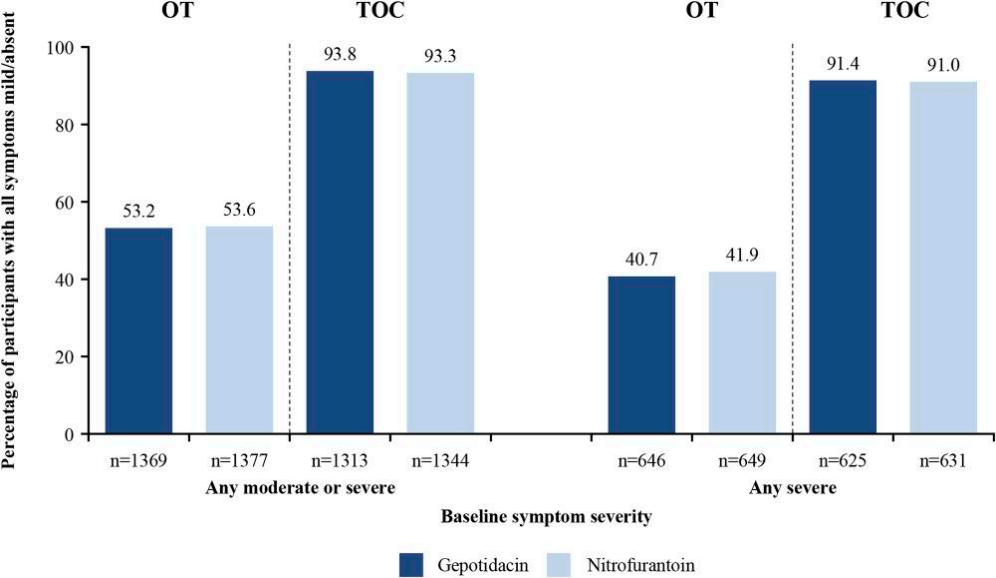
Participants with symptoms no longer interfering with daily activities (mild/absent) at OT and TOC visits by baseline symptom severity (post-hoc analysis; pooled ITT population). Abbreviations: ITT, intent-to-treat; OT, on-therapy; TOC, test-of-cure.

Among participants with any baseline symptoms interfering with or preventing everyday activities (moderate or severe) in the pooled ITT population, approximately 53% in both treatment groups reported that symptoms no longer affected everyday activities (ie, all mild or absent) at the OT visit (Figure 4). Among participants with severe baseline symptoms preventing everyday activities, approximately 41% reported only mild or absent symptoms at the OT visit. In both treatment groups, the proportion of participants with symptoms no longer interfering with daily activities generally increased by the study day of the OT visit (Supplementary Figure 2). By TOC, symptoms had resolved or no longer affected everyday activities for >90% of participants across treatment arms in these subgroups (Figure 4). Symptom severity data at OT and TOC stratified by severity at baseline are presented in Supplementary Table 6.

## DISCUSSION

Alleviation of urinary symptoms with effective treatment of uUTI is fundamental to patients' well-being [4], and early symptom improvement is a key outcome measure to consider when assessing the efficacy of treatments for uUTI. However, data on early symptom improvement are limited, as registrational trials focus on composite endpoints at fixed post-treatment timepoints, missing the impact of earlier symptom relief.

The current analysis investigated pooled symptom response data from two Phase 3, randomized, double-blind trials (EAGLE-2 and EAGLE-3). In the pooled ITT population, early clinical response rates (symptom resolution or improvement) at OT were similar between treatments (gepotidacin, 81.4%; nitrofurantoin, 82.1%). As might be expected, the proportion of participants classified as early clinical responders increased across the OT visit window (Day 2 and Day 5: 73.7% and 90.5%, respectively, for gepotidacin; and 71.8% and 98.6%, respectively, for nitrofurantoin). By the TOC visit, a large proportion of participants reported full symptom resolution (66.5% gepotidacin; 64.0% nitrofurantoin) with an additional 22.1% (gepotidacin) and 25.9% (nitrofurantoin) reporting improvement from baseline. Clinical outcomes at TOC among participants in the pooled ITT population were similar to those among participants with a culture-confirmed bacterial infection (≥10^5^ CFU/mL and comparator-susceptible at baseline) and across the individual studies, which were previously reported [18]. Among the four urinary symptoms assessed (dysuria, frequency, urgency, and lower abdominal pain), symptom relief was broadly similar across the individual symptoms in both treatment groups.

While the study entry criteria were designed to exclude participants with chronic urinary symptoms that could interfere with symptom measurements and/or preclude complete resolution of uUTI symptoms, some participants reported a history of non-infectious urinary symptoms in the prior 12 months. In a sensitivity analysis, removing these participants, clinical outcomes at the OT and TOC visits were similar to the overall ITT population. This finding was not surprising given that these participants constituted just 9% of those enrolled.

A symptom score threshold that represents clinically meaningful symptom relief has not been validated for uUTI; therefore, a post-hoc analysis of functional symptom improvement was conducted. This analysis showed that among participants with urinary symptoms affecting everyday activities at baseline (moderate or severe), >53% of participants treated with either gepotidacin or nitrofurantoin reported that symptoms no longer affected everyday activities (mild or absent) at the OT visit, and >93% of participants reported that symptoms no longer affected everyday activities by the TOC visit.

While the OT visit data reported herein illustrate that symptoms are improved with gepotidacin and nitrofurantoin before the completion of treatment, rapid reduction in uUTI symptom severity has been reported in other studies (non-registrational) that measured symptom improvement at earlier time points than those of the OT visits in EAGLE-2 and EAGLE-3. In one study, 50% of participants treated with single-dose ciprofloxacin (*N* = 264) experienced symptom improvement (measured by the Urinary Tract Infection Symptom Assessment Questionnaire) by 6 hours after dosage [12]. In another study, the median time to symptom improvement with ciprofloxacin was 2.4 days among participants responsive to treatment [20]. Booth et al reported that among a small cohort (n = 41) of participants given trimethoprim, more than half (54%) reported a symptom duration of 2 days or less [21]. Time to symptom improvement or resolution was not assessed in EAGLE-2 or EAGLE-3 as participants did not attend daily visits, and Day 2 was the earliest time point examined. Nonetheless, as large, randomized controlled trials, EAGLE-2 and EAGLE-3 provide a robust data set for exploration of early symptom responses.

Examination of all enrolled participants in EAGLE-2 and EAGLE-3 (ie, 3136 participants in the pooled ITT population) offers a broader patient population than the primary analysis populations of each trial, improving generalizability of the results. Symptom scoring was used to provide a more objective measure of treatment effects on uUTI symptoms compared to investigator-assessed clinical response; though these still had a subjective component and require validation for future use as a measure of treatment success. The study designs were based specifically on the FDA guidelines for uUTI clinical trials [9], in which the definition of uUTI may differ from other guideline definitions. Caution should, therefore, be taken in applying these findings to uUTI as defined by different criteria.

Study limitations should be acknowledged. Early clinical response at OT was assessed over several days (the protocol-defined visit window, study Days 2–4, was widened for analysis to include Day 5 to utilize out-of-window data considered consistent with the time point). While most participants completed the OT visit on Day 2 (30%) or Day 3 (37%), some completed the visit later. For this reason, early clinical response rates were explored by study day of the completed OT visit in a post-hoc analysis. Study visits were scheduled at the screening/baseline visit; however, participants were not randomly allocated to specific days and attended a single visit while on therapy and at TOC. Therefore, comparisons across study days should be treated with caution. Additionally, the timing of relief from individual symptoms with either treatment could not be assessed with this study design; however, no notable differences were apparent based on rates of individual symptom improvement at OT and resolution at TOC. At the time of writing, additional research with gepotidacin is ongoing to further characterize the time course of early symptom improvement, assessing individual participant symptoms daily during treatment (NCT06597344) [22, 23].

In the analysis of clinical outcomes, participants with missing data were treated as non-responders (at OT) and clinical failures (at TOC); although common practice in registrational trials, this may have resulted in under-estimation of treatment responses. Clinical outcome/response analyses in the CE population addressed this limitation by excluding participants with missing outcome data; however, because CE populations are defined by protocol compliance (ie, participant inclusion is based on events that occur post-randomization), these analyses are subject to potential post-randomization bias. Nonetheless, treatment differences for both early clinical response and clinical success (resolution) at TOC were similar in the pooled ITT and CE populations.

Finally, a limitation of our early response analysis was that any symptom score reduction was considered an improvement, but this may not reflect a meaningful benefit to quality of life. Our analysis of functional symptom improvement was conducted to address this limitation.

## CONCLUSIONS

Gepotidacin and nitrofurantoin provide similar early relief of uUTI symptoms. In patients that need an alternative option to standard-of-care treatment, gepotidacin may be an option that enables them to resume normal daily activities shortly after initiating treatment. This is the first study to thoroughly evaluate early response in uUTI, which is an important outcome for patients. Early response should be prioritized as an important outcome in future studies.

## Supplementary Material

ciaf722_Supplementary_Data

## Supplementary Data

Supplementary materials are available at *Clinical Infectious Diseases* online. Consisting of data provided by the authors to benefit the reader, the posted materials are not copyedited and are the sole responsibility of the authors, so questions or comments should be addressed to the corresponding author.
