# Supplementary material for: Early Symptom Response in Patients With Uncomplicated Urinary Tract Infection Treated With Gepotidacin or Nitrofurantoin: Pooled Analysis From Two Pivotal Phase 3 Studies
Source: Clin Infect Dis. 2026 Jan 6;82(4):e684–92. doi: 10.1093/cid/ciaf722 (PMC13131927; doi:10.1093/cid/ciaf722)
Supplement: ciaf722_Supplementary_Data [file ciaf722_supplementary_data.docx]

**Supplementary materials**

**Supplementary Table 1.** Baseline symptom severity (post hoc analysis; ITT population)

| **Baseline symptom scores** | **Gepotidacin**  **1500 mg BID**  **N=1572** | **Nitrofurantoin**  **100 mg BID**  **N=1564** | **Total**  **N=3136** |
| --- | --- | --- | --- |
| Any moderate or severe symptom | 1440 (92%) | 1445 (93%) | 2885 (92%) |
| Any severe symptom | 678 (43%) | 678 (43%) | 1356 (43%) |
| Worst symptom was moderate | 762 (49%) | 767 (49%) | 1529 (49%) |
| No moderate or severe symptoms | 131 (8%) | 117 (7%) | 248 (8%) |

Data are n (%); percentages were calculated with non-missing data as the denominator. One gepotidacin group participant and two nitrofurantoin group participants were missing baseline assessments for all individual symptom domains.
BID, twice daily; ITT, intent-to-treat.

**Supplementary Figure 1.** Visit completion by study day (post hoc analysis; ITT population)


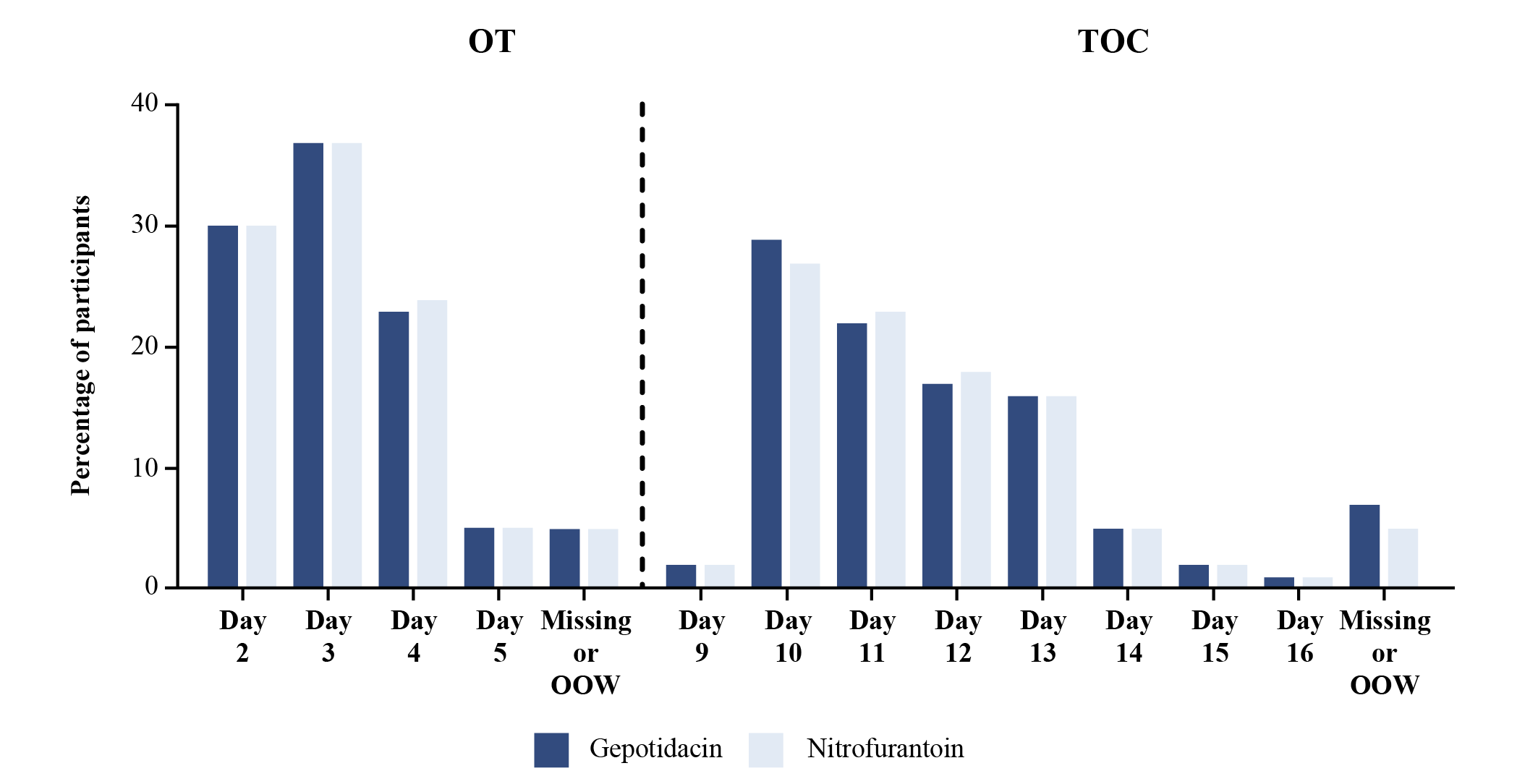


Number of participants: 1572 gepotidacin group, 1564 nitrofurantoin group
ITT, intent-to-treat; OOW, out-of-window; OT, on-therapy; TOC, test-of-cure.

**Supplementary Table 2.** Early clinical response (improvement/resolution) at on-therapy visit by Study Day (post hoc analysis; pooled ITT population)

| **Study day of OT visit** | **Gepotidacin**  **1500 mg BID**  **N=1572** | **Nitrofurantoin**  **100 mg BID**  **N=1564** |
| --- | --- | --- |
| **Day 2** | 351/476 (73.7%) | 333/464 (71.8%) |
| **Day 3** | 512/575 (89.0%) | 528/583 (90.6%) |
| **Day 4** | 340/362 (93.9%) | 352/371 (94.9%) |
| **Day 5** | 76/84 (90.5%) | 71/72 (98.6%) |

Data are n/N1 (%), where N1 is the number of participants who completed their OT visit on the specified study day. Each study participant attended a single OT visit, which was scheduled at the screening/baseline visit.
BID, twice daily; ITT, intent-to-treat; OT, on-therapy.

**Supplementary Table 3.** Clinical outcome and response by visit (pooled CE populations)

| **Visit (population)**  **Clinical response**  **Clinical outcome** | **EAGLE-2 and EAGLE-3 pooled results** | | |
| --- | --- | --- | --- |
|  | **Gepotidacin**  **1500 mg BID** | **Nitrofurantoin**  **100 mg BID** | **Difference (95% CI)** |
| **On-therapy (CE-OT)** | **N=1401** | **N=1429** |  |
| Early clinical response* | 1217 (86.9) | 1239 (86.7) | –0.2 (–2.6, 2.2) |
| Clinical resolution | 146 (10.4) | 144 (10.1) |  |
| Clinical improvement (without resolution) | 1071 (76.4) | 1095 (76.6) |  |
| Non-response (worsening/no change) | 184 (13.1) | 190 (13.3) |  |
| **Test-of-cure (CE-TOC)** | **N=1386** | **N=1424** |  |
| Clinical success (resolution) | 1017 (73.4) | 980 (68.8) | 4.7 (1.4, 8.1) |
| Clinical failure | 369 (26.6) | 444 (31.2) |  |
| Clinical improvement (without resolution) | 332 (24.0) | 394 (27.7) |  |
| Clinical worsening (or no change) | 37 (2.7) | 50 (3.5) |  |

Data are n (%) unless otherwise stated. Treatment difference (gepotidacin – nitrofurantoin) and 95% CIs were calculated using the Miettinen-Nurminen method adjusted for study. Clinical outcomes for participants who took other systematic antibacterials for uUTI prior to or on the same date as their clinical assessment were set to ‘clinical worsening’ (OT: 0 [0%] gepotidacin group, 1 [<0.1%] nitrofurantoin group; TOC: 20 [1.4%] gepotidacin group, 38 [2.7%] nitrofurantoin group). *Data are from post-hoc analyses.
BID, twice daily; CE, clinically evaluable; CI, confidence interval; OT, on-therapy; TOC, test-of-cure; uUTI, urinary tract infection.

**Supplementary Table 4.** Sensitivity analysis of the clinical outcome and response by visit (excluding participants with non-uUTI urinary symptoms in the prior 12 months) (post hoc analysis; ITT population)

| **Visit**  **Clinical response**  **Clinical outcome** | **EAGLE-2 and EAGLE-3 pooled results** | | |
| --- | --- | --- | --- |
|  | **Gepotidacin**  **1500 mg BID**  **N=1416** | **Nitrofurantoin**  **100 mg BID**  **N=1436** | **Difference (95% CI)** |
| **On-therapy** |  |  |  |
| Early clinical response | 1156 (81.6) | 1181 (82.2) | –1.1 (–3.8, 1.7) |
| Clinical resolution | 137 (9.7) | 129 (9.0) |  |
| Clinical improvement (without resolution) | 1019 (72.0) | 1052 (73.3) |  |
| Non-response | 260 (18.4) | 255 (17.8) |  |
| Clinical worsening (or no change) | 192 (13.6) | 196 (13.6) |  |
| Unable to determine | 68 (4.8) | 59 (4.1) |  |
| **Test-of-cure** |  |  |  |
| Clinical success (resolution) | 963 (68.0) | 942 (65.6) | 2.4 (–1.1, 5.8) |
| Clinical failure | 453 (32.0) | 494 (34.4) |  |
| Clinical improvement (without resolution) | 297 (21.0) | 361 (25.1) |  |
| Clinical worsening (or no change) | 52 (3.7) | 63 (4.4) |  |
| Unable to determine | 104 (7.3) | 70 (4.9) |  |

Data are n (%) unless otherwise stated. Treatment difference (gepotidacin – nitrofurantoin) and 95% CI were calculated using the Miettinen-Nurminen method adjusted for study. Clinical outcomes for participants who took other systematic antibacterials for uUTI prior to, or on the same date as, their clinical assessment were set to ‘clinical worsening’.

BID, twice daily; CI, confidence interval; OT, on-therapy; TOC, test-of-cure; uUTI, uncomplicated urinary tract infection.

**Supplementary Table 5:** Symptom improvement (OT) and resolution (at TOC) by individual symptom present at baseline (post-hoc analysis; pooled ITT population)

|  | **Gepotidacin**  **1500 mg BID** | **Nitrofurantoin**  **100 mg BID** |
| --- | --- | --- |
| **OT** |  |  |
| Dysuria improved | 979/1474 (66.4) | 982/1443 (68.1) |
| Frequency improved | 967/1518 (63.7) | 977/1506 (64.9) |
| Urgency improved | 977/1463 (66.8) | 1011/1453 (69.6) |
| Lower abdominal or suprapubic pain improved | 887/1269 (69.9) | 896/1295 (69.2) |
| **TOC** |  |  |
| Dysuria resolved | 1156/1474 (78.4) | 1117/1443 (77.4) |
| Frequency resolved | 1132/1518 (74.6) | 1102/1506 (73.2) |
| Urgency resolved | 1153/1463 (78.8) | 1133/1453 (78.0) |
| Lower abdominal or suprapubic pain resolved | 1038/1269 (81.8) | 1055/1295 (81.5) |

Data are n/N1 (%) where N1 is the number of participants with specified symptom present at baseline (score>0). Symptom improvement/resolved categories required no antibacterial use for uUTI prior to the post-baseline assessment but were defined regardless of other systemic antibacterial use (i.e., not for uUTI).
BID, twice daily; ITT, intent-to-treat; OT, on-therapy; TOC, test-of-cure visit; uUTI, uncomplicated urinary
tract infection. Data are from post-hoc analyses.

**Supplementary Figure 2.** Participants with symptoms no longer interfering with daily activities (mild/absent) at OT visit by study day (post hoc analysis; pooled ITT population)


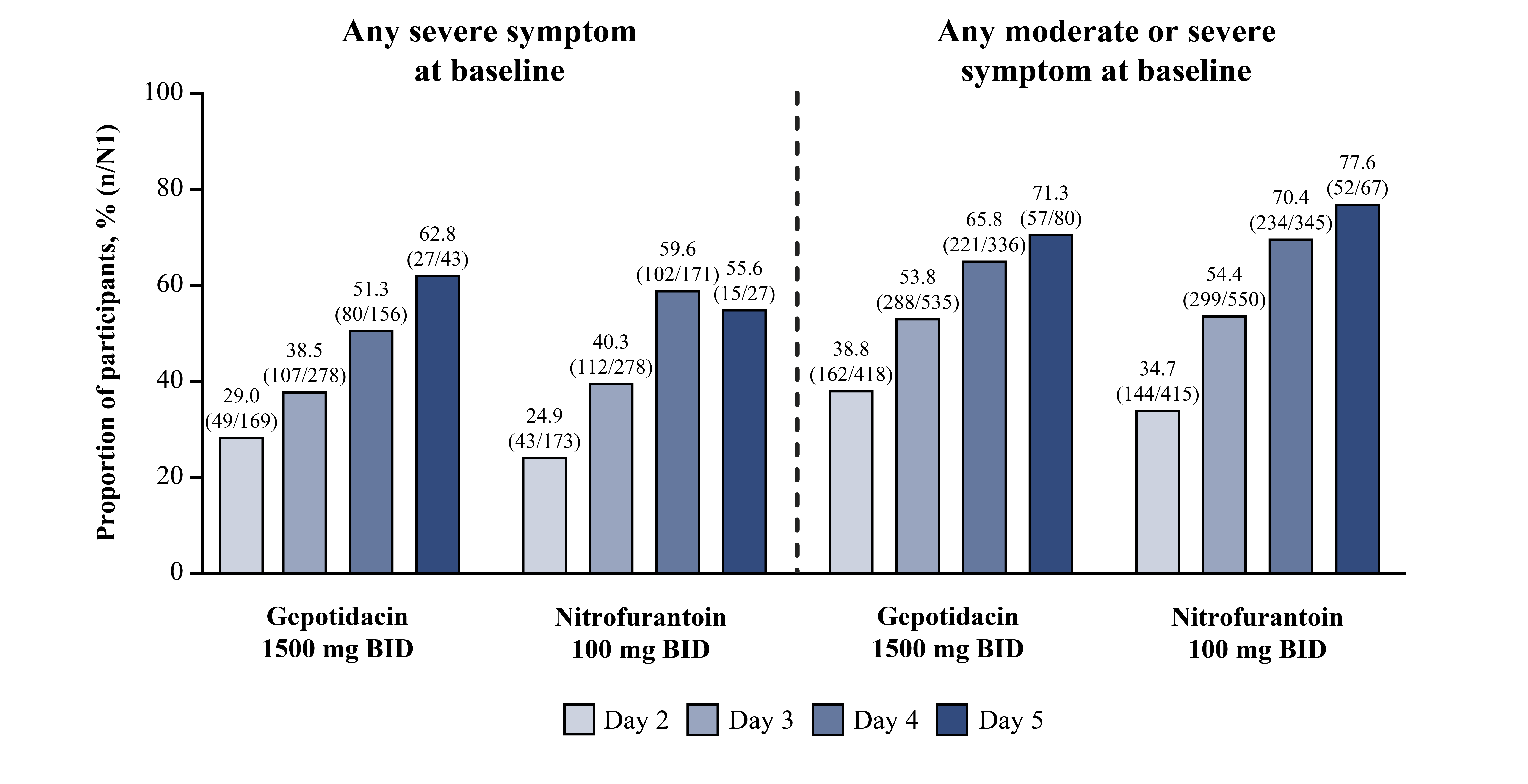


N1 is the number of participants with the noted baseline symptom severity who completed their OT visit on each day and who also had non-missing baseline and post-baseline symptom assessments, without use of non-study systemic antibacterials for uUTI prior to the symptom assessment. n is the number of these participants with all symptoms recorded as mild/absent. BID, twice daily; OT, on-therapy; ITT, intent-to-treat; uUTI, uncomplicated urinary tract infection.

**Supplementary Table 6.** Symptom severity at OT and TOC stratified by severity at baseline (post hoc analysis; ITT population)

| **Visit** | **Baseline symptom severity** | **Post baseline symptom severity, n (%)** | **Gepotidacin**  **1500 mg BID** | **Nitrofurantoin**  **100 mg BID** |
| --- | --- | --- | --- | --- |
| OT | Any severe symptom (any individual  score = 3) | Participants included, N1 | 646 | 649 |
|  |  | Any severe symptoms | 78 (12.1) | 83 (12.8) |
|  |  | Any moderate symptoms; none severe | 305 (47.2) | 294 (45.3) |
|  |  | All symptoms mild/absent | 263 (40.7) | 272 (41.9) |
|  |  | All symptoms absent | 50 (7.7) | 47 (7.2) |
|  |  | Participants excluded from analysis, N2 | 32 | 29 |
|  |  | Systemic AB received for uUTI prior to assessment | 3 | 0 |
|  |  | Missing post-baseline symptom assessment | 29 | 29 |
|  | Any moderate or severe symptom (any individual score = 2 or 3) | Participants included, N1 | 1369 | 1377 |
|  |  | Any severe symptoms | 91 (6.6) | 88 (6.4) |
|  |  | Any moderate symptoms; none severe | 550 (40.2) | 551 (40.0) |
|  |  | All symptoms mild/absent | 728 (53.2) | 738 (53.6) |
|  |  | All symptoms absent | 135 (9.9) | 126 (9.2) |
|  |  | Participants excluded from analysis, N2 | 71 | 68 |
|  |  | Systemic AB received for uUTI prior to assessment | 7 | 1 |
|  |  | Missing post-baseline symptom assessment | 64 | 67 |
|  | No moderate or severe symptoms (individual scores ≤1) | Participants included, N1 | 121 | 112 |
|  |  | Any severe symptoms | 0 | 0 |
|  |  | Any moderate symptoms; none severe | 9 (7.4) | 8 (7.1) |
|  |  | All symptoms mild/absent | 112 (92.6) | 104 (92.9) |
|  |  | All symptoms absent | 25 (20.7) | 23 (20.5) |
|  |  | Participants excluded from analysis, N2 | 10 | 5 |
|  |  | Systemic AB received for uUTI prior to assessment | 0 | 0 |
|  |  | Missing post-baseline symptom assessment | 10 | 5 |
| TOC | Any severe symptom (any individual  score = 3) | Participants included, N1 | 625 | 631 |
|  |  | Any severe symptoms | 10 (1.6) | 15 (2.4) |
|  |  | Any moderate symptoms; none severe | 44 (7.0) | 42 (6.7) |
|  |  | All symptoms mild/absent | 571 (91.4) | 574 (91.0) |
|  |  | All symptoms absent | 430 (68.8) | 400 (63.4) |
|  |  | Participants excluded from analysis, N2 | 53 | 47 |
|  |  | Systemic AB received for uUTI prior to assessment | 6 | 12 |
|  |  | Missing post-baseline symptom assessment | 47 | 35 |
|  | Any moderate or severe symptom (any individual  score = 2 or 3) | Participants included, N1 | 1313 | 1344 |
|  |  | Any severe symptoms | 12 (0.9) | 20 (1.5) |
|  |  | Any moderate symptoms; none severe | 69 (5.3) | 70 (5.2) |
|  |  | All symptoms mild/absent | 1232 (93.8) | 1254 (93.3) |
|  |  | All symptoms absent | 949 (72.3) | 912 (67.9) |
|  |  | Participants excluded from analysis, N2 | 127 | 101 |
|  |  | Systemic AB received for uUTI prior to assessment | 21 | 24 |
|  |  | Missing post-baseline symptom assessment | 106 | 77 |
|  | No moderate or severe symptoms (individual scores ≤1) | Participants included, N1 | 119 | 112 |
|  |  | Any severe symptoms | 1 (0.8) | 1 (0.9) |
|  |  | Any moderate symptoms; none severe | 1 (0.8) | 1 (0.9) |
|  |  | All symptoms mild/absent | 117 (98.3) | 110 (98.2) |
|  |  | All symptoms absent | 100 (84.0) | 93 (83.0) |
|  |  | Participants excluded from analysis, N2 | 12 | 5 |
|  |  | Systemic AB received for uUTI prior to assessment | 1 | 1 |
|  |  | Missing post-baseline symptom assessment | 11 | 4 |

N1 is the number of participants with non-missing baseline and post-baseline symptom assessments without use of non-study systemic antibacterials for uUTI prior to the symptom assessment; N2 is the number of participants with non-missing baseline assessments and post-baseline symptom assessments that were missing or confounded by prior non-study systemic antibacterial use for uUTI.

AB, antibacterial; BID, twice daily; ITT, intent-to-treat; OT, on-therapy visit; TOC, test-of-cure visit; uUTI, uncomplicated urinary tract infection.

**Alternate text**

**Supplementary Figure 1:** Bar graph showing the visit completion by study day at OT and TOC for gepotidacin and nitrofurantoin. The timing of post-baseline OT visits completed was similar between treatment groups**.**

**Supplementary Figure 2:** Bar graph showing the proportion of participants with symptoms no longer interfering with daily activities by study day of the On-Therapy visit among participant subgroups with any moderate or severe symptom at baseline or any severe symptom at baseline. The percentage of patients with symptoms no longer interfering with daily activities increased across study days.
